# Complete Genome Sequence of Stenotrophomonas indicatrix DAIF1

**DOI:** 10.1128/MRA.01484-20

**Published:** 2021-02-11

**Authors:** Ines Friedrich, Jacqueline Hollensteiner, Janna Scherf, Judith Weyergraf, Anna Klassen, Anja Poehlein, Robert Hertel, Rolf Daniel

**Affiliations:** a Genomic and Applied Microbiology and Göttingen Genomics Laboratory, Institute of Microbiology and Genetics, University of Göttingen, Göttingen, Germany; b FG Synthetic Microbiology, Institute of Biotechnology, BTU Cottbus-Senftenberg, Senftenberg, Germany; University of Southern California

## Abstract

We present the complete genome of Stenotrophomonas indicatrix DAIF1, which was isolated from an oligotrophic pond in a water protection area. Whole-genome alignments indicated that strain DAIF1 belongs to the species Stenotrophomonas indicatrix. The whole genome (4,639,375 bp) harbors 4,108 protein-encoding genes, including 3,029 genes with assigned functions.

## ANNOUNCEMENT

Stenotrophomonas indicatrix DAIF1 was isolated from an oligotrophic pond water sample from Germany (51°33′58″N, 9°56′22″E) as described previously (1). The genome of environmentally derived strain DAIF1 is of interest for comparative genome analysis with clinical isolates. For genomic DNA preparation, the strain was cultured in PCa medium (peptone medium supplemented with 0.015% CaCl_2_) at 30°C (2). DNA was purified with the MasterPure complete DNA and RNA purification kit as recommended by the manufacturer (Epicentre, Madison, WI, USA). The isolated DNA was used to generate Illumina sequencing libraries using the Nextera XT DNA sample preparation kit and was sequenced on a MiSeq instrument with reagent kit v3 (2 × 300 bp, 600 cycles) as recommended by the manufacturer (Illumina, San Diego, USA). For sequencing with the MinION system, the 1D genomic DNA sequencing protocol in combination with the ligation sequencing 1D kit (SQK-LSK109) and the native barcode expansion kit (EXP-NBD103; barcode 11) were used as recommended by the manufacturer (Oxford Nanopore Technologies, Oxford, UK). Input DNA without size selection was end repaired with NEBNext FFPE repair mix (New England Biolabs, Ipswich, MA, USA). Nanopore sequencing was performed by using the SpotON flow cell Mk I (R9.4.1) for 72 h with MinKNOW software v18.12.6. Guppy v3.4.1 was employed in fast mode for demultiplexing and base calling. Default parameters were used for all software unless otherwise specified. Nanopore and Illumina reads were quality processed with fastp v0.19.5 (3), resulting in 35,352 Nanopore reads with sizes ranging from 10 to 50 kbp (*N*_50_, 14.5 kbp) and 2,256,826 high-quality Illumina paired-end reads. A Nanopore long-read assembly with the racon v1.3.1 assembler as part of the Unicycler pipeline v0.4.7 (4) resulted in a single circular chromosome with a final coverage of 214-fold. Sequence polishing was performed using the Illumina reads and the unicycler_polish.py script (4); this resulted in a final genome of 4,639,375 bp with a GC content of 66.36%. Prokka v1.13.3 (5) was used for automatic annotation, which resulted in 4,108 protein-encoding genes, of which 3,029 were assigned functions. Furthermore, 76 tRNA genes, 1 transfer-messenger RNA gene, and 13 rRNA genes were identified.

**FIG 1:**
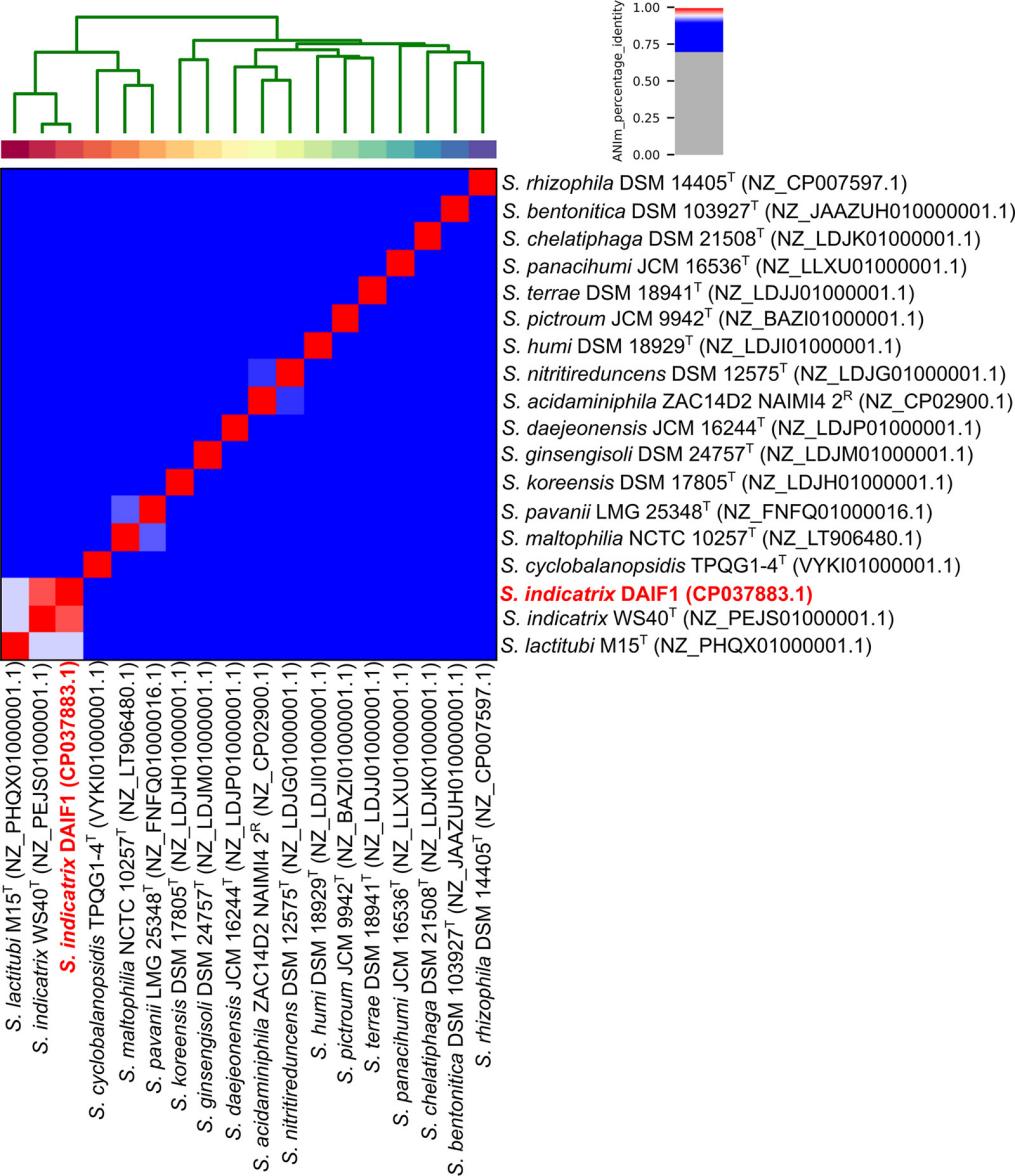
ANI analysis of the Stenotrophomonas indicatrix DAIF1 genome (red). All available genomes of type strains (superscript T) and representative strains (superscript R) from the genus *Stenotrophomonas* were taken into account. Calculations were performed with the Python pyANI package (9) using the ANIm (ANI calculated by using a MUMmer3 implementation) method with standard parameters. Analysis revealed a genome sequence identity of 98.29% for DAIF1 in comparison with the *S. indicatrix* WS40^T^ genome. GenBank accession numbers are provided in parentheses.

In order to provide a first phylogenetic classification, a BLAST search against the NCBI nonredundant nucleotide database using the 16S rRNA gene sequence of DAIF1 was performed (6). The most similar 16S rRNA gene was from *Stenotrophomonas* sp. strain MYb57 (GenBank accession number KU902436.1), with an identity of 100%. To further classify DAIF1, its genome was compared with all available genomes of *Stenotrophomonas* type strains (Fig. 1) by using average_nucleotide_identity.py v0.2.10 (https://github.com/widdowquinn/pyani) with the option ANIm and MUMmer3 (7). Average nucleotide identity (ANI) analysis (8) showed that DAIF1 clustered with *S. indicatrix* WS40^T^ (GenBank accession number NZ_PEJS00000000.1). The recorded identity was 98.39% (Fig. 1). This is higher than the species boundary of approximately 94% (9) and allows assignment of DAIF1 as a new strain within the *S. indicatrix* species. Furthermore, the closed genome sequence of *S. indicatrix* DAIF1 is beneficial for comparative genomics with clinical isolates.

### Data availability.

The annotated genome sequence of *Stenotrophomonas* sp. DAIF1 and the 16S rRNA gene sequence were submitted to GenBank under the accession numbers CP037883 and MW078496, respectively. Raw reads were deposited in the NCBI Sequence Read Archive (SRA) under the accession numbers SRX6039405 (Nanopore reads) and SRX6039404 (Illumina reads).
